# LONGEVITY IN THE PACIFIC: FINDINGS FROM THE KUAKINI HAWAII CENTENARIAN STUDY

**DOI:** 10.1093/geroni/igae098.0382

**Published:** 2024-12-31

**Authors:** Bradley Willcox, Kamal Masaki, Peter Martin, Gina Lee, Hardeep Obhi, Leonard Poon

**Affiliations:** Kuakini Medical Center, Honolulu/Kuakini Medical Center, Hawaii, United States; Kuakini Medical Center, Honolulu/Kuakini Medical Center, Hawaii, United States; Iowa State University, Ames, Iowa, United States; University of Wisconsin Madison, Madison, Wisconsin, United States; University of California Davis, Davis, California, United States; University of Georgia, Athens, Georgia, United States

## Abstract

The immigration of Japanese citizens to Hawaii more than one hundred years ago profoundly changed the culture of these islands. Their diet, exercise, and spirituality may have influenced the longevity of Hawaii residents. This presentation highlights longevity predictors among Hawaiian men of Japanese descent. A sample of 3,734 participants (Mage = 77.82 years at baseline) from the Kuakini Hawaii Centenarian Study was included in this research. With follow-up longitudinal data, we compared participants who survived into their 70s, their 80s, their 90s, or to 100 years of age. Results indicated that cognitive function, F(3,3,733) = 52.59, p <.001, and self-reported health, F(3,3,733) = 18.72, p <.001, at baseline distinguished those who survived to centenarian status compared to those who died in their 70s, 80s, or 90s. Centenarians were more likely to carry the protective FOXO3 allele, χ2 (N=3,352) = 8.94, p <.05, and less likely to be diagnosed with diabetes, χ2 (N=3,560) = 12.43, p <.01. Regression analyses suggested that father’s age death (β =.04, p <.05), fluid intelligence (β =.12, p <.001), ADL impairment (β = -.23 p <.001), health behaviors, (i.e., BMI, smoking and drinking, β =.55, p <.01, β = -.10, p <.001, and β = -.08, p <.01, respectively), and diabetes (β = -.06, p <.01), were significantly associated with age at death. The results support policymakers and practitioners in promoting healthy longevity among a culturally distinct group.

